# Testing the effects of two different zebrafish exposure paradigms on transcriptomic-based chemical risk assessment using the flame retardant triphenyl phosphate

**DOI:** 10.1093/toxsci/kfaf124

**Published:** 2025-09-09

**Authors:** Michael G Morash, Morgan W Kirzinger, John C Achenbach, Ananda B Venkatachalam, Joseph P M Hui, Susanne Penny, Kevin Stemmler, Joëlle Pinsonnault Cooper, Deborah E Ratzlaff, Cindy L A Woodland, Lee D Ellis

**Affiliations:** Aquatic and Crop Resource Development, National Research Council of Canada, Halifax, NS B3H 3Z1, Canada; Aquatic and Crop Resource Development, National Research Council of Canada, Saskatoon, SK S7N 0W9, Canada; Aquatic and Crop Resource Development, National Research Council of Canada, Halifax, NS B3H 3Z1, Canada; Aquatic and Crop Resource Development, National Research Council of Canada, Halifax, NS B3H 3Z1, Canada; Aquatic and Crop Resource Development, National Research Council of Canada, Halifax, NS B3H 3Z1, Canada; Human Health and Therapeutics, National Research Council of Canada, Halifax, NS B3H 3Z1, Canada; Aquatic and Crop Resource Development, National Research Council of Canada, Halifax, NS B3H 3Z1, Canada; New Substances Assessment and Control Bureau, Health Canada, Ottawa, ON K1A 0K9, Canada; New Substances Assessment and Control Bureau, Health Canada, Ottawa, ON K1A 0K9, Canada; New Substances Assessment and Control Bureau, Health Canada, Ottawa, ON K1A 0K9, Canada; Aquatic and Crop Resource Development, National Research Council of Canada, Halifax, NS B3H 3Z1, Canada

**Keywords:** zebrafish embryo toxicity, general behavioral toxicity, transcriptomics, triphenyl phosphate, zebrafish

## Abstract

In the zebrafish larval toxicity model, phenotypic changes induced by chemical exposure can potentially be explained and predicted by the analysis of gene expression changes at sub-phenotypic concentrations. The increase in knowledge of gene pathway-specific effects arising from the zebrafish transcriptomic model has the potential to enhance the role of the larval zebrafish as a component of Integrated Approaches to Testing and Assessment (IATA). In this paper, we compared the transcriptomic responses to triphenyl phosphate between 2 standard exposure paradigms, the Zebrafish Embryo Toxicity (ZET) and General and Behavioral Toxicity (GBT) assays. The ZET assay represents a developmental model with chemical exposure from 6 to 120 h post fertilization (hpf), which covers organogenesis, whereas the GBT represents a juvenile model with exposure from 72 to 120 hpf, which occurs post-organogenesis. This comparison demonstrates both similarities and differences between the 2 assays. Although both models identified similar xenobiotic metabolism pathways, the difference in exposure window length and the time of transcriptomic sampling between the 2 methods also yielded unique sets of affected pathways, demonstrating their complimentary nature. Both data sets support previously described effects of triphenyl phosphate on aquatic and mammalian systems. This work validates and strengthens the use of both exposure paradigms and continues to demonstrate that zebrafish larvae are a valuable tool in the context of IATA toward reduced reliance on the use of higher vertebrate derived data for chemical risk assessment.

As the potential role of zebrafish larvae in chemical risk assessment is becoming more recognized, it is critical that the many advantages of the model be integrated to maximize its predictive potential. The larval zebrafish has recently been validated for the identification of reproductive and developmental toxicity for pharmaceuticals, demonstrating the relevance of this model in risk assessment (Weiner et al. 2024). Similarly, larval zebrafish transcriptomics are becoming a powerful tool for the elucidation of the mechanism of action of known toxicants (Wang and Liu 2023). Although transcriptomics offer insight into mechanisms of toxicity, the effect of the chemical exposure paradigm must be determined to help ensure that data derived from these studies are optimally informative for risk assessors. A number of factors can potentially influence the organismal response, including dose (Suvorov 2024), uptake/metabolism (Achenbach et al. 2022; Kossack et al. 2023), and growth stage (Liu et al. 2024). In addition, the complexity and amount of data generated by these approaches make holistic evaluation of the data difficult (Mlecnik et al. 2018). Similarly, seemingly disparate groups of ontologies may share commonalities that are not apparent during manual curation of the data.

Triphenyl phosphate (TPP, TPhP or TPHP) is an organophosphate flame retardant and plasticizer currently in use as a replacement for the increasingly banned polybrominated diphenyl ethers (National Toxicology Program 2018). Although there is evidence for TPP-induced neurotoxicity (Hawkey et al. 2023; Zhang et al. 2023), TPP is a suspected endocrine-disrupting chemical affecting various aspects of metabolism (Beausoleil et al. 2024), reproduction, and development (Witchey et al. 2023). With respect to aquatic organisms, TPP displays considerable toxicity (Chen et al. 2022). We have previously reported differences in phenotypic and behavioral outcomes for two different zebrafish larval exposure paradigms (Achenbach et al. 2020). The first exposure paradigm involves the exposure of larvae from 6 to 120 h post fertilization (hpf; Zebrafish Embryo Toxicity—ZET) and the second an exposure from 72 to 120 hpf (General and Behavioral Toxicity—GBT). Both assays revealed cardiac toxicity/edema and body positioning/morphology deficits, which have been described previously after exposure to TPP (Fan et al. 2022; Wiegand et al. 2023). In addition, the GBT assay showed liver toxicity and behavioral effects not seen in the ZET assay (Achenbach et al. 2020). Although behavioral effects are well documented for other flame retardants, whether TPP causes behavioral changes is unclear. TPP has been shown to cause hypoactivity in a 6-day developmental exposure paradigm, and this is enhanced when doing acute (<24 h) exposure (Jarema et al. 2015). Conversely, in a comprehensive study of flame retardants and toxicity end points, TPP was shown to induce what are likely non-specific behavioral effects (Alzualde et al. 2018).

In 2024, due to its endocrine-disrupting properties, TPP was added to the European Union’s (EU’s) Candidate List of substances of very high concern for authorization published in accordance with Article 59(10) of the Registration, Evaluation, Authorization and Restriction of Chemicals (REACH) Regulation (ECHA 2024b). TPP promotes estrogenic activity through Cyclin D1 and stimulating cell cycle progression (Kwon et al. 2022). In adult zebrafish, TPP exposure affects expression of Estradiol (E2), Vitellogenin, and several genes in the Hypothalamus/Pituitary/Gonad axis in a sex-specific way (Liu et al. 2013b). TPP exposure resulted in decreased body length, and at high concentrations (72 to 96 hpf exposure 10 μM+) caused changes in insulin-like growth factor (*igf-1*), thyroid hormone receptor α (*trα*), transthyretin (*ttr*), thyroid-stimulating hormone β (*tshβ*), iodothyronine deiodinase 1 (*dio1*), but no estrogenic activity (*cyp19a1b*) (Lee et al. 2022). ZET-like exposure to ∼6 μM TPP induced expression of genes related to the aryl hydrocarbon receptors, peroxisome proliferator-activated receptor (PPAR)-α, and thyroid receptor, glucocorticoid, and mineralocorticoid receptor pathways (Liu et al. 2013a). TPP affects T3/T4 levels and related gene expression in zebrafish at 7 dpf (from 3 hpf) (Kim et al. 2015).

In this paper, we combine toxicokinetic profiling of TPP exposure with RNAseq to demonstrate exposure paradigm differences in the toxicity profiles. Specifically, we describe the similarities and differences in transcriptomic responses induced by TPP between the ZET and GBT exposure paradigms. Gene ontology (GO)-term enrichment analysis revealed that though detoxification pathways were transcriptionally active in both exposure paradigms, important differences in multiple processes (i.e. multiple pathways related to DNA replication/chromosome maintenance and several endocrine disruption-related genes) were identified only using the GBT assay. This illustrates the importance of sub-phenotypic transcriptomic profiling in the development of larval zebrafish models as an alternative for animal-derived data.

## Materials and methods

### Chemicals

TPP (CAS Registry Number 115-86-6, purity ≥99%) and DMSO (Cat#D8418, purity ≥ 99%) were purchased from Sigma-Aldrich (Oakville, ON, Canada). TPP was dissolved in DMSO at a stock concentration of 200 mM. All stocks were stored at −20°C.

### Animal husbandry

All animal husbandry was performed as previously described (Morash et al. 2023, 2024). Briefly, age-matched zebrafish embryos (AB/Tub hybrids) were collected in E3 media (5 mM NaCl, 0.17 mM KCl, 0.33 mM CaCl_2_–2H_2_O, 0.33 mM MgSO_4_–7H_2_O) and stored in 10 × 150 mm disposable polystyrene petri dishes at 28.5°C. Embryos for GBTs were then transferred in groups of 200 to nursery baskets (Pentair Aquatic Ecosystem, Apopka, FL, USA) in a 3-l tank on a ZebTec Recirculation Water Treatment System (Tecniplast USA, Easton, PA, USA) and raised until 72 hpf. The recirculation system was housed in a room kept on a 14:10-h light:dark cycles, water temperature maintained at 28.5 ± 0.5°C. Embryos for ZET assays were transferred to HE3 media (E3 + 10 mM HEPES, pH 7.2). All procedures were performed in accordance with the Canadian Council of Animal Care (CCAC) guidelines.

### Molecular uptake assay

Chemical exposures were based on the ZET and GBT models, with embryos and larvae exposed to each chemical from 6 to 120 hpf and 72 to 120 hpf, respectively. Larvae were exposed to each test compound at a single concentration based on their EC_20_ values (ZET: 2.27 µM, GBT: 4.10 µM) as previously published (Achenbach et al. 2020). Briefly, at 6 hpf for ZET and 72 hpf for GBT, fertilized embryos were placed singly into the well of a 96-well polystyrene square-well microplate (Biolite, Fisher Scientific, ON, Canada) in 270 µl of HEPES-buffered E3 media (5 mM NaCl, 0.17 mM KCl, 0.33 mM CaCl_2_–2H_2_O, 0.33 mM MgSO_4_–7H_2_O). A 10× final exposure concentration stock (ZET: 22.7 µM, GBT: 41.0 µM, both 5% DMSO in HE3) was made prior to each exposure; 30 µl of the 10× solution was pipetted into each well to obtain EC_20_ concentrations and start the exposure (final DMSO concentration 0.5% [v/v]). Plates were sealed with ThermoSeal RTS clear-transparent film (Excel Scientific, Victorville, CA, USA) to prevent evaporation and incubated at 28.5°C on a 14:10 light:dark cycle (light intensity 3 to 5 µmol m^−2^ s^−1^). At the desired time points (2, 18, 42, 66, 114 h of exposure for ZET and 2, 6, 24, and 48 h of exposure for GBT), 20 viable (≤24 hpf absence of coagulation, ≥48 hpf presence of heartbeat) embryos–larvae were quickly removed from each well and placed into a 74 mm Netwell (Corning, USA) sitting in a dry 10 × 150 mm polystyrene petri dish. A 1 ml media sample (ZET) or 100 µl sample (GBT) was removed and placed in an amber 2 ml liquid chromatography (LC) vial and placed at −80°C for flash freezing. Embryos–larvae were then washed by immersion in 4 ml of ice-cold carrier media 4 times. After the final wash step, the Netwells, were immersed in 4 ml of ice-cold water. The 20 larvae were transferred to a 2 ml amber LC vial in a minimal amount of water (ZET) or to a Lysis Prep 2 ml tube containing Lysing Matrix D (MP Biomedicals, Solon, OH, USA), and the water was removed using a gel-loading tip (GBT). The vials were then placed at −80°C until later processing. For the 0 h exposure samples, the embryos–larvae were washed and placed at −80°C immediately after addition of the test compound. A “media-only” parallel control experiment was performed with no larvae added to the 270 µl of HE3 media, and 30 µl of the same 10× stock solution was added. Plates containing these controls were sealed and housed in identical incubation conditions. The control media samples were removed at the same time points as those containing larvae. The 1 ml volume of the “media-only” samples was obtained by collecting 200 µl directly from 5 wells for the ZET-style exposure and 100 µl from a single well for the GBT-style exposure. These were immediately placed at −80°C for storage. For percent recovery determination, 20 age-matched larvae incubated in carrier control (CC) media under the same conditions were sampled, washed as above, and, for the ZET-style exposure, placed in a 2 ml LC vial containing 60 µL of the same 10× compound stock solution used in the corresponding molecular uptake experiment. This was then placed at −80°C for storage. For the GBT-style exposures, the percent recovery control samples were made by adding 7.5 µl of a 0.2 mM TTP solution in DMSO to each Lysis Matrix D vial containing the 20 age-matched larvae.

### Extraction of larval and media samples

For the ZET-style exposure samples, frozen larvae and media samples were lyophilized directly in the LC vials. Lyophilization occurred at a shelf temperature at −5°C for 24 h and then 0°C for additional 48 h. Lyophilized samples were extracted by adding 1 ml of methanol and placed in a sonicating water bath for 30 to 45 min to disrupt tissue and enhance solubilization by the methanol. Samples were clarified before liquid chromatography-high resolution mass spectrometry (LC-HRMS) analysis by spinning at 3000 × *g* for 30 min at 4 °C with transfer of the supernatant to a clean 2 ml LC vial. For the GBT-style exposure samples, the media samples that were collected directly into LC vial were thawed and diluted with methanol to a final volume of 1 ml. Samples were then clarified before LC-HRMS analysis by spinning at 750 × g for 30 min at 4 °C with transfer of the supernatant to a clean 2 ml LC vial for LC-HRMS analysis; 1 ml of methanol was added to the Lysis Prep tubes containing larvae, and the larvae were disrupted using a Fast Prep 24 instrument (MP Biomedicals) for 40 s at a speed of 6.0 m/s. Lysate was then transferred to a 2 ml LC vial gel loading tip and the lysate spun at 750 × *g* for 30 min at 4 °C. Supernatant was then transferred to 2 ml amber LC vials for subsequent LC-HRMS analysis. All extracts were stored at −20 °C.

### LC-HRMS analysis

LC-HRMS was performed on an UltiMate^TM^ 3000 LC pump coupled to a Q Exactive™ Orbitrap mass spectrometer (Thermo Fisher Scientific, Waltham, MA, USA), equipped with an HESI-II probe for electrospray ionization. Methanol extracts were separated on a Waters BEH-C18 column (2.1 × 100 mm, 1.7 µm, Waters, Milford, MS, USA) using a mobile phase of (A) 0.1% formic acid in water and (B) 0.1% formic acid in acetonitrile. Analytes were eluted with a linear gradient of 40% B to 100% B in 4 min, held for 3.3 min, before returning to 5% B. The LC flow rate was kept at 400 µl/min, whereas the column temperature was set at 30 °C. Optimized MS parameters including a sheath gas flow rate of 50, auxiliary gas flow rate of 10, spray voltage of 3.0 kV, capillary temperature of 350°C, and heater temperature of 250 °C were used. Positive polarity scans were acquired at 3 Hz (70,000 instrument resolution), with mass range scanned from m/z 90 to 1000. Data acquisition was carried out using Xcalibur 4.4 (Thermo Fisher Scientific, Waltham, MA, USA).

The peak areas of TPP and its hydroxylated metabolite were extracted from the MS chromatogram, with 5 ppm mass windows centered on the accurate masses, to calculate their concentrations. In order to account for matrix effects, 2 standard curves ranged from 0.01 to 2.5 µM were generated. One was used for larval extracts as a diluent for the methanol extract of 120 hpf-aged larvae exposed to CC media and processed as in the molecular uptake assay. The second was used for media extracts as a diluent for the methanol extract from the corresponding media, again processed as in the molecular uptake assay.

### Calculations

#### Percent recovery correction

All values were corrected by the result of the percent recovery experiment from an age-matched embryo/larval sample. For media extracts, the time 0 h no-larvae media only sample was used to determine percent recovery for the extraction procedure.

#### Toxicant uptake

Equation (1) **pmol per embryo** = [Corrected larval extract concentration in µM] × [1 L/1000 ml] × [1000,000 pmol/µmol] × [1/20 larvae]

### Transcriptomic sampling

Transcriptomic sampling and sequencing were performed as described previously (Morash et al. 2023). Briefly, either 6 hpf (ZET) or 72 hpf (GBT) larvae were individually transferred to a 96-round-well microtiter plate in 270 µl of HE3 medium. A 10× EC_20_ final exposure concentration stock (see Molecular Uptake Assay section above for 10× stock composition) was prepared and serially diluted 10-fold to make stock solutions for transcriptomic exposures; 30 µl of each 10× stock was then added to 24 wells to generate the 10-fold series of exposures; 0.5% DMSO was used as a CC. Plates were sealed using ThermoSeal RTS clear-transparent film (Excel Scientific, Victorville CA, USA) and incubated at 28.5°C without media replenishment. At 120 hpf (114 h [ZET] or 48 h [GBT]) of exposure, 20 phenotypically normal larvae were collected into Corning Netwell baskets and rinsed 5 times in HE3 + 0.5% DMSO, followed by once in type I Milli-Q water and collected into microcentrifuge tubes. Residual water was removed and larvae were flash frozen on dry ice and stored at −80°C. Exposure experiments were performed 3 separate times to derive the triplicate samples used in these analyses.

### RNA isolation and sequencing

Total RNA was isolated using Norgen total RNA columns (Norgen Cat#17200, Thorold, ON, Canada) followed by on-column DNase I digestion (Norgen Cat#25710, Thorold, ON, Canada). Total RNA was quantitated by spectroscopy with average recoveries of TPP ZET: 244.84 µg/ml (27.60 to 497.53), and TPP GBT: 298.80 µg/ml (239.04 to 366.32). RIN values were determined (Agilent RNA 6000 pico kit, Mississauga, ON, Canada), and RIN values averaged 8.7. Sequencing libraries were prepared using 1 µg of total RNA (Illumina Truseq Stranded mRNA kit, San Diego, CA, USA) and sequenced on an Illumina Hiseq 2500 using High Output V4 2 × 125 bp chemistry. Library sizes averaged ∼15 M reads per sample. Reads were aligned to the zebrafish genome (GRCz11) using STAR under default parameters (Dobin et al. 2013). Approximately 83% of all reads were uniquely mapped to the genome, whereas 12.9% of reads mapped to multiple loci, and ∼3.5% of reads were not mapped. Sequencing data were uploaded to the NCBI Gene Expression Omnibus repository (GSE291969). Selected alignment statistics are shown in Table S1.

### Differential gene expression analysis

Raw reads were processed as described previously (Morash et al. 2023). DESeq2 (Love et al. 2014) was used for all differential gene expression (DEG) analyses using a fold change (FC) of 1.5 (also represented as the log_2_ of FC = 0.585) and an adjusted *P*-value false discovery rate of 0.05 (Benjamini–Hochberg correction). Results of pairwise comparisons for the ZET and GBT TPP experiments are shown in Files S1 and S2, respectively. Volcano plots were generated using R (R Core Team 2022). Heatmaps ([Kolde 2012] pheatmap: Pretty Heatmaps. R package version 1.0.12) were made using the Pearson correlation distance measure, with a normalized count threshold of 25, and graphed as log2 (EC_20_/CC) with no row scaling.

### GO analysis

DEGs identified by DESeq2 (CC vs EC_20_) above were subjected to GO-term/KEGG analysis using ClueGO (Bindea et al. 2009). Default parameters were used, including 4 GO ontologies (Biological process, Molecular function, cellular component, and immune system process) and KEGG. GO term fusion and a *P*-value cutoff of 0.05 was applied with a Benjamini–Hochberg correction. The complete results are included in File S3.

### Benchmark dose curve fitting and point of departure calculations

Benchmark Dose (BMD) curve fitting was done as described (Morash et al. 2023, 2024), using normalized quantile data from DESeq2, imported into BMDExpress 2.3 (Phillips et al. 2019). A Williams Trend Test (1.5-fold cut off) was used, and all fitting models were used. Post-filtering to remove BMDU/BMDL > 40, BMDs > the EC_20_ concentration, and BMDs < 10-fold below the lowest tested concentration. Transcriptomic Point of Departure (tPOD) using the 10th percentile of BMD values was calculated, and the first mode by analyzing for modes and anti-modes using a custom R script (Page-Lariviere et al. 2019). Mode graphs are shown in Fig. S1.

## Results

### Uptake analysis

**Fig. 1.**
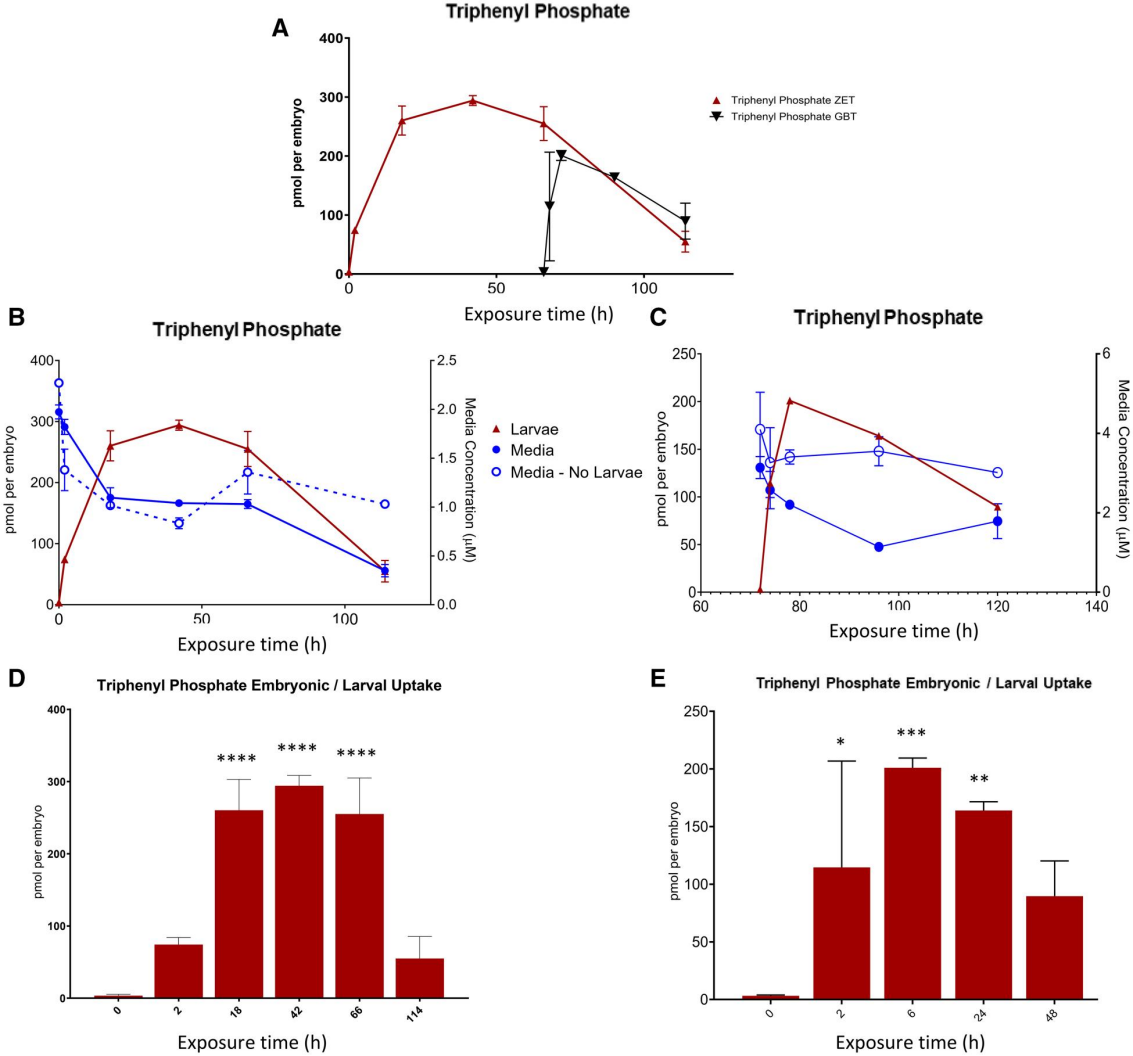
Toxicokinetic analysis of TPP under ZET (6 to 120 hpf) and GBT (72 to 120 hpf) exposure paradigms (A). Embryonic/larval uptake of TTP is expressed as pmol per embryo over the time course of exposure. TPP was measured in embryos/larvae, media, and media alone in ZET (B) and GBT (C) exposure protocols. All values are the mean of 3 experiments with error bars denoting standard deviation. Lines shown are trendlines and do not represent a fitted equation. Larval uptake of TPP at each of the tested exposure time points is compared with the levels at time 0 of the ZET (D) and GBT (E) exposures using a one-way ANOVA with a Dunnett’s multiple comparisons test. *, **, ***, **** = *P* < 0.05, 0.01, 0.001, and 0.0001, respectively.

Exposure to TPP in the ZET assay resulted in a peak larval uptake of 294.1 pmol per embryo at 48 hpf, followed by a decrease to 58.4 pmol per larva by 120 hpf (Fig. 1A, B, and D). There was also a concomitant decrease in the TPP concentration in the media during exposure (Fig. 1B). Control experiments demonstrated stability of TPP in the absence of embryos/larvae (Fig. 1B). In the GBT exposure paradigm, in which exposure begins at 72 hpf, the peak uptake (200.9 pmol per larva) occurred 6 h after initiation of exposure (Fig. 1A, C, and E). As with the ZET assay, media levels of TPP decreased during the exposure and remained stable in the absence of larvae (Fig. 1C).

### Transcriptomic analysis

**Fig. 2.**
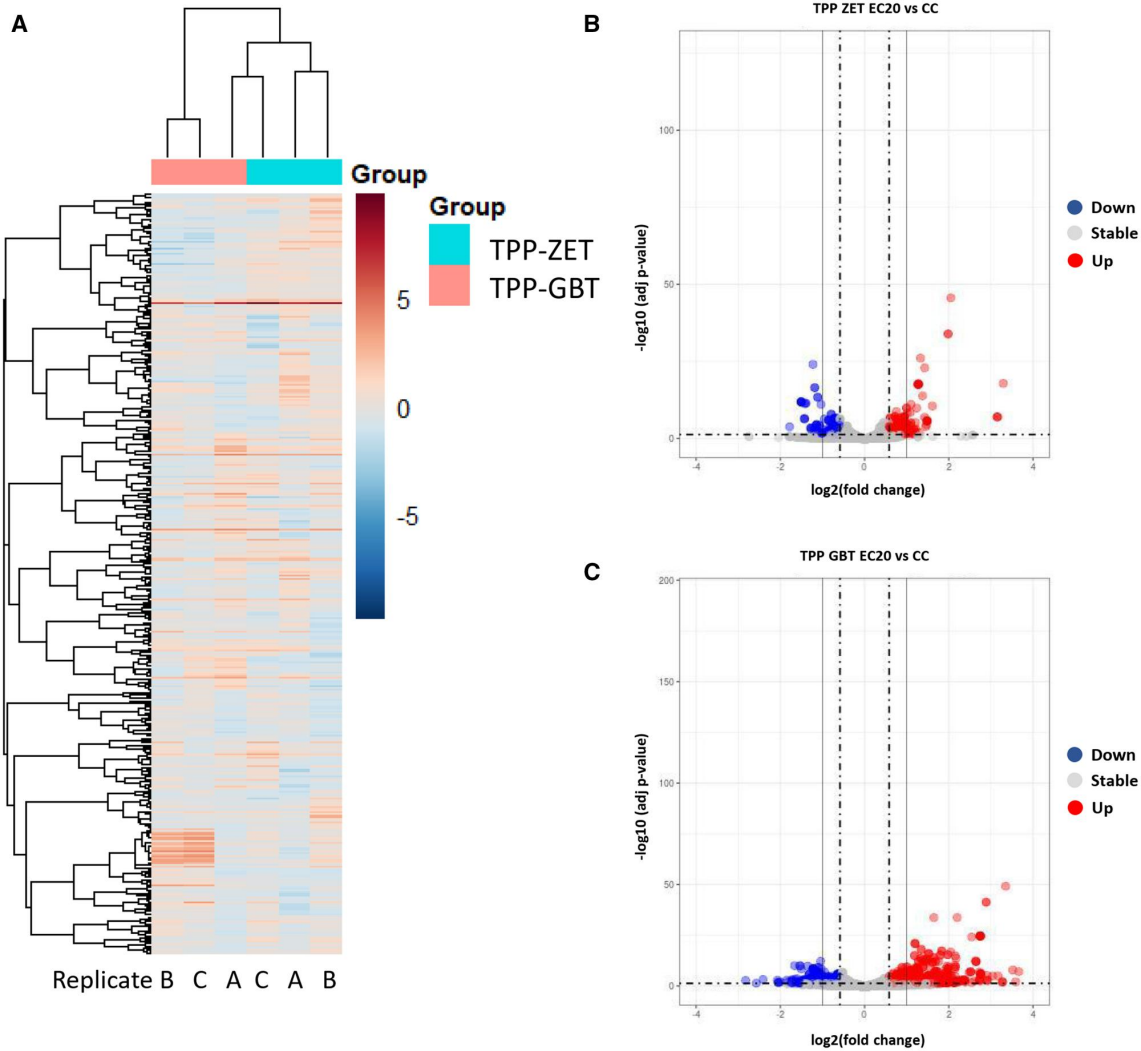
Differentially expressed genes (DEGs) in TPP identified in the ZET and GBT assays. (A) Heatmap of triplicate (A, B, and C) TPP samples. (B and C) Volcano plots showing distribution of up- (red) and downregulated (blue) genes upon TPP exposure in the ZET (B) and GBT (C) assays.

TPP induced a number of gene expression changes in both the ZET and GBT assays. Specifically, using our cutoff criteria for differentially expressed genes, exposure to TPP under the ZET paradigm produced 211 DEGs (81 downregulated, 130 upregulated, Fig. 1). The GBT assay produced more DEGs than the ZET assay, with 887 genes (367 upregulated, 520 downregulated, Fig. 2). When comparing DEGs between the 2 paradigms, 107 were common to both. Of the 107 common DEGs, 106 (99%) remained either up- or downregulated in both assays, with only one (*pdzk1*) changing from down- (ZET) to upregulated (GBT), indicating conservation of the effects of TPP in the paradigms.

A limited number of endocrine-related genes (*star*, *pgrmc1*) were identified among the ZET assay DEGs. Conversely, in addition to those found in the ZET assay, a number of DEGs were identified during the GBT exposure that are related to estrogen/progesterone, obesogenic, and thyroid function (Table 1).

**Table 1.** List of endocrine-related DEG’s identified in the GBT assay.

	Gene name	FC	P-adj	Function
Estrogen/progesterone	*pagr1*	−1.799	0.04331	Estrogen receptor binding
	*hsd11b2*	1.567	0.001165	Steroid synthesis
	*hsd17b14*	1.683	0.02963	Steroid synthesis
	*pgrmc1*	1.852	7.12E-10	Receptor binding
	*star*	2.108	0.046239	Steroid synthesis
	*porb*	2.279	9.33E-16	Steroid synthesis
	*elf3*	2.619	4.03E-10	Estrogen receptor regulator
	*hpxa*	9.679	0.009972	Cellular response to estrogen
Obesity	*fto*	−1.609	0.039764	Insulin/obesity
	*steap4*	1.540	0.009956	Obesity
	*fstl3*	1.560	0.013282	Adipose tissue
	*igfbp1a*	1.653	0.001433	Insulin
	*pnpla3*	1.853	0.001028	Obesity/fatty acid
	*me1*	2.403	0.002433	Obesity
	*ephx1*	2.456	9.41E-08	Diabetes
Thyroid	*dio1*	1.772	0.011857	Thyroid hormone synthesis
	*dio3b*	1.863	0.01297	Thyroid hormone synthesis
	*pth1a*	2.372	0.019667	Thyroid function

Negative values indicate downregulated genes.

LFC, log2 of fold change; *P*-adj, adjusted *P*-value.

BMD modeling revealed very similar tPOD (mode) values for the ZET (0.014 µM) and GBT (0.025 µM) assays, both values being ∼100 times lower than their respective EC_20_ values. The POD (mode) values were identical to the POD 10th values. ClueGO analysis of the DEGs making up the POD (mode) in the ZET and GBT assays did not produce any significant GO/KEGG-terms.

### GO analysis of EC_20_ vs CC DEGs

**Fig. 3.**
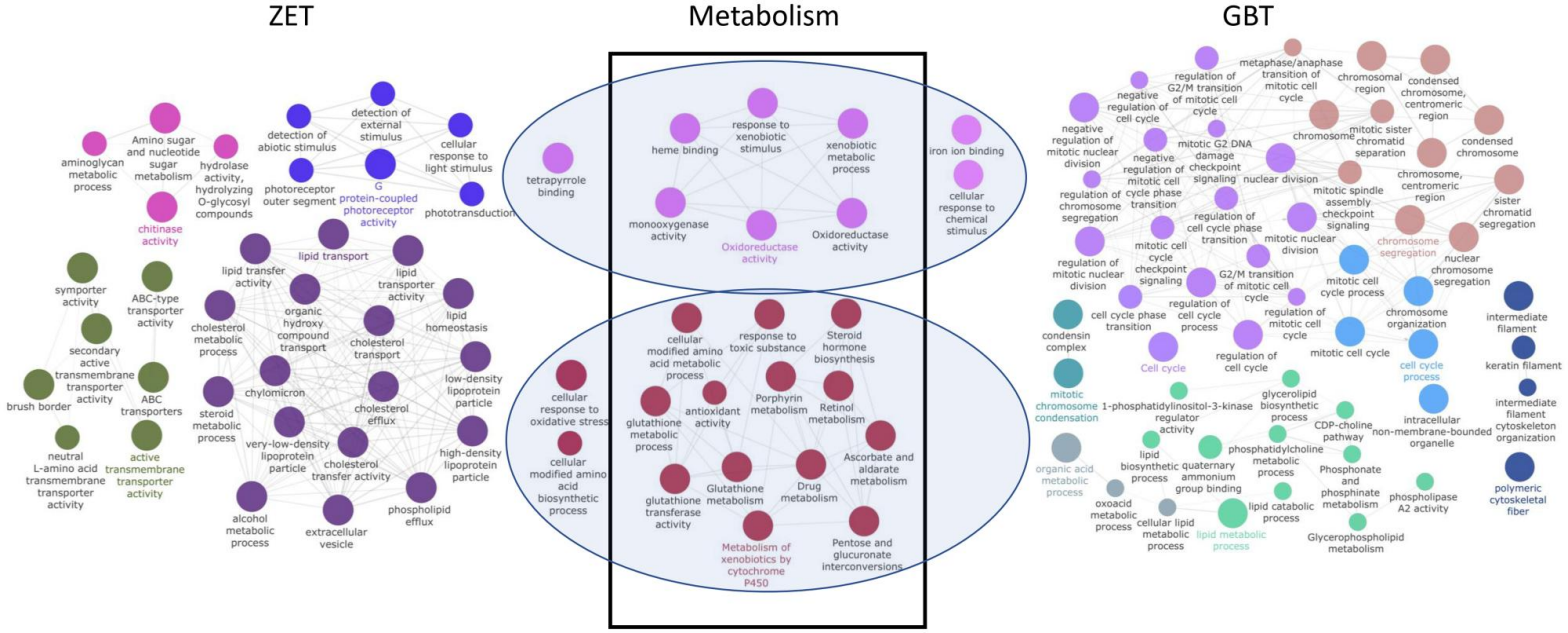
GO analysis of ZET and GBT exposure DEGs.

The DEGs identified above were subjected to GO analysis using the Cytoscape application ClueGO (Fig. 3). The ZET assay produced terms related to lipid transport, membrane transport, and chitinase activity. The GBT produced terms related to cell cycle, chromosomes, and lipid metabolism. The 107 DEGs common to both exposure paradigms were then subjected to GO-term analysis to identify shared pathways. Interestingly, these shared DEGs were related mainly to detoxification and drug metabolism. The top 10 significant GO/KEGG terms identified by ClueGO for each exposure paradigm are shown in Table 2.

**Table 2.** Top 10 significant GO/KEGG terms identified by ClueGO.

Tabular results of GO analysis.					
ID	Term	Ontology source	Group *P*-value	# Genes	Example genes
GO:0016705	Oxidoreductase activity	GO: MF	8.90E-09	13	*bbox1, cyp26a1, cyp2y3, fads2, phyh*
GO:0022804	Active transmembrane transporter activity	GO: BP	1.04E-08	17	*abcb11b, abcb5, abcc2, abcg2a, slco1d1*
GO:0006575	Cellular-modified amino acid metabolic process	GO: BP	2.14E-06	7	*bbox1, gch1, gsto1, gstp2, gstr*
GO:0006869	Lipid transport	GO: BP	2.17E-06	13	*afp4, apoea, ldlrb, star, ttpa*
GO:0004364	Glutathione transferase activity	GO: MF	2.28E-06	5	*gsto1, gstp2, gstr, gstt1b, mgst3b*
GO:0015036	Disulfide oxidoreductase activity	GO: MF	2.94E-03	3	*glrx, gsto1, txn*
GO:0004568	Chitinase activity	GO: MF	3.10E-03	3	*chia.1, chia.2, chia.6*
GO:0008020	G protein-coupled photoreceptor activity	GO: BP	4.65E-03	4	*opn1lw2, opn1mw1, opn1sw2, rho*
GO:0000793	Condensed chromosome	GO: CC	7.90E-03	5	*bub1, incenp, ncapd2, nuf2, smc2*
KEGG:00980	Metabolism of xenobiotics by cytochrome P450	KEGG	8.60E-12	10	*ephx1, gsto1, gstp2, mgst3b, ugt1a1*

GBT					
ID	Term	Ontology source	Group *P*-value	# Genes	Example genes
GO:0022402	Cell cycle process	GO: BP	6.17E-16	66	*cdca8, chek1, kif11, ncaph, rad50*
GO:0016712	Oxidoreductase activity	GO: MF	2.55E-08	16	*cyp2aa12, cyp2aa4, cyp2ad2, cyp2k16, cyp8b1*
GO:0020037	Heme binding	GO: MF	3.51E-08	29	*cyb5a, cyp3a65, hbae3, mb, ptgesl*
GO:0007059	Chromosome segregation	GO: BP	2.79E-07	37	*birc5a, cenpf, nek2, top2a, tprb*
GO:0007076	Mitotic chromosome condensation	GO: BP	9.19E-06	6	*ncapd2, ncaph, nusap1, smc2, smc4*
GO:0006629	Lipid metabolic process	GO: BP	9.52E-06	57	*abhd4, chpt1, pck1, socs3a, star*
GO:0006082	Organic acid metabolic process	GO: BP	1.54E-05	45	*acbd7, cyp2aa12, lpl, ptgesl, tdo2a*
GO:0099513	Polymeric cytoskeletal fiber	GO: CC	1.80E-05	34	*cenpe, lmna, nefmb, numa1, tpx2*
KEGG:00983	Drug metabolism	KEGG	3.41E-10	20	*cdab, gstp2, tk1, ugt1a1, xdh*
KEGG:00591	Linoleic acid metabolism	KEGG	1.90E-05	7	*cyp2p8, cyp3c4, pla2g12b, pla2g1b, rarres3l*

## Discussion

In this paper, we demonstrate that an assessment of the uptake kinetics of a compound, along with the profiling of transcriptomic data, is an important contribution to an integrated approach that strengthens the zebrafish larval toxicity platform. In determining the uptake kinetics, it is possible to identify not only whether a chemical is taken up by the larvae but also evaluate the potential bioaccumulation, metabolism, and excretion of the compound. These factors can all be critical considerations in the assessment of toxicity. We do recognize that the single maximal peaks for each assay are a consequence of static exposure conditions, and that the results may be different under a renewal exposure system. However, our data demonstrated no issues with larval uptake of TPP, and given the previously reported confounding effects attributed to media renewal (injury, larval loss, desiccation) (Hamm et al. 2019), we chose to proceed with a static exposure for both experimental paradigms.

In this work, we demonstrate the value of assessing toxicity using our GBT assay, which uses a later developmental exposure window than the standard OECD FET 236 assay. It is important to note that there is overlap between the ZET and GBT exposure paradigms, with the GBT (72 to 120 hpf) assay falling within the exposure time of the ZET (6 to 120 hpf) assay. Apart from the obvious differences in developmental stages between the 2 assays, the presence/absence of a chorion represents a significant difference. Chorion permeability has been described as a limitation in embryonic zebrafish toxicity testing by others (Gustafson et al. 2012; Kim and Tanguay 2014). Specifically, the presence of a chorion has been shown to obscure tail and spine damage phenotypes caused by exposure to TPP and the behavioral effects of other organophosphate esters (Tran et al. 2021). Similarly, hatching defects can produce phenotypes that are unrelated to the toxicity of the chemical (Mandrell et al. 2012). Premature removal of the chorion allows chemical accessibility but necessarily deviates from natural development (Ninness et al. 2006; Thomas et al. 2009) and introduces the possibility of injury or death through additional handling steps. By using naturally hatched larvae, the GBT assay avoids chorionic complications that may affect uptake in the FET/ZET assay. Initiation of the static exposure at the later (72 hpf) time point may also help explain the differences from the ZET assay we observed. Exposure during earlier time points may have allowed for the expression of compensatory mechanisms or the lack of target gene expression at the most sensitive developmental period. This highlights the importance of exposure paradigm considerations in toxicity testing.

The EU recently classified TPP as a Substance of Very High Concern because of endocrine-disrupting properties, especially with respect to fish (ECHA 2024a). We identified a number of endocrine-related genes induced by TPP exposure in the GBT assay, related to estrogen/progesterone, obesity, and thyroid function (Table 1). We describe several changes to thyroid-related gene expression, including deiodinases and parathyroid hormone 1a, which supports previous findings that the goitrogenic activity of TPP at early stages of zebrafish development suggests potential endocrine-disrupting activity (Kim et al. 2015). TPP alters the expression of several thyroid-related genes in zebrafish larvae (Lee et al. 2022) and also causes sex-specific thyroid disruption in adult zebrafish (Liu et al. 2019). Development of the zebrafish thyroid gland is not complete until 72 hpf (Marelli and Persani 2017), at which time TPP levels have started to decrease in the ZET assay and may no longer be at levels sufficient to induce thyroid gene-expression changes. We also identified several obesogenic/metabolic-related genes after GBT exposure, including effects on lipid and cholesterol homeostasis. In human cell lines, TPP exposure causes increases to intracellular insulin and proinsulin levels (Pavlíková et al. 2024) and induces a number of lipid effects (An et al. 2023). In fetal mice, TPP exposure causes multiple metabolic defects, including insulin resistance and increased body weight (Wang et al. 2019a). In adult zebrafish, TPP causes lipid and carbohydrate metabolism perturbations in the liver (Du et al. 2016). In this work, we demonstrate a number of gene expression changes in steroidogenic and estrogen receptor-related genes. In support of our findings, sex hormone endocrine-disruptive activities have also been described in larval zebrafish upon exposure to organophosphate esters (Zhang et al. 2024), azoxystrobin (Jiang et al. 2018), and norethindrone (Luo et al. 2020) as measured by gene expression changes. Whether the findings in the current study support perturbations in specific axes and whether these have potential effects during adult development will require further evaluation. However, we feel it is important to note that evaluation of a complex, multi-organ system such as the HPG axis is greatly facilitated by the use of a whole-organism model, and as such, provides information for hazard assessment that may not be obvious from less complex systems. We also identified potential effects on progesterone signaling (*pgrmc1*). Interestingly, progesterone signaling is critical during the development of dopaminergic neurons (Willing and Wagner 2016), and it is tempting to speculate alterations in dopaminergic neurons may account for the behavioral alterations reported after TPP exposure in the GBT assay (Achenbach et al. 2020). Indeed, it has been suggested that TPP exposure may lead to general defects in nervous system development (Alzualde et al. 2018), and changes in several neurotoxicity transcripts have been described (Shi et al. 2018). The significance of these DEGs in only the GBT assay is unclear but of interest. We are aware that the TPP induced expression of endocrine-related genes in the GBT assay may not be broadly applicable in all cases, but the notion that endocrine disruption may be better evaluated at later points should not be overlooked. It is possible that the exposure kinetics in the GBT assay are more favorable to inducing endocrine-related genes than the ZET. The timing of exposure has been shown to coincide when certain receptors are expressed, as may be the case with PPAR-γ and TPP (Garoche et al. 2021).

For this work, we analyzed the gene set lists using ClueGO, which builds non-redundant, functionally organized networks and identifies representative pathways (Bindea et al. 2009). In the context of an early life-stage zebrafish New Approach Method, consolidating large sets of transcriptomic data into information required by those conducting chemical risk assessments is critical. Our goal is to use the most enriched pathways to predict possible apical toxicity endpoints. By comparing the transcriptomic results and identifying significantly enriched biological pathways, which may be indicative of toxicological mechanisms of action, we hope to predict possible apical toxicity endpoints. The most significant pathways identified in the GBT exposures were related to chromosome function and DNA replication/integrity. Using hepatocellular cell lines exposed to TPP, Xie et al. (2023) demonstrated micronucleus formation and chromosome damage in a *cyp*-dependent manner. Similarly, Du et al. (2016) demonstrated changes in cell cycle, DNA replication, and repair in the livers of adult zebrafish. TPP has also been shown to stimulate cell-cycle progression in endometrial cells (Kwon et al. 2022). We identified a number of cell cycle-related genes affected, indicating this is a predominant effect of TPP exposure on zebrafish larvae in the GBT assay. It is possible that these effects would have been seen in the ZET assay if transcriptomic sampling had been performed using a similar exposure window (i.e. 48 to 72 hpf). It is reasonable to assume that these effects are limited to a narrow time window, given that multiple days of cell-cycle and DNA repair perturbations would be expected to have significant deleterious effects. Importantly, in a regulatory context, alterations in DNA repair and cell cycle function could be considered an indication of genotoxicity and justification for following up with genotoxicity tests within an Integrated Approaches to Testing and Assessment.

TPP exposure using the ZET paradigm also produced unique GO/KEGG terms. Specifically, the ZET assay identified a number of photoreceptor-related GO terms. It has been reported that TPP downregulates opsin expression in a FET-like exposure paradigm (2 to 144 hpf) (Shi et al. 2019) and causes down-regulation of genes involved in eye development (perception, retina) (Zhang et al. 2020). Lipid dysregulation, another significant set of GO/KEGG terms identified in the ZET assay, has been well described. Increased cholesterol and triglyceride content, along with lipid accumulation, have been reported in HepG2 cells after TPP exposure (An et al. 2023). Early exposure to TPP has also been shown to reduce levels of neutral lipids in zebrafish larvae (Reddam et al. 2019). More frequent transcriptomic sampling, especially at points closer to the peak internal concentration of TPP in the ZET assay, may reveal whether these changes are immediate (i.e. induced directly by TPP) or are the result of downstream perturbations. Although zebrafish-based transcriptomic testing continues to be a powerful tool for elucidating mechanisms of toxicity, the field of transcriptomic-based regulatory toxicology is still very much in development, and this work, which highlights the value of assessing multiple exposure paradigms in the context of a whole-organism, multi-system approach to assessing toxicity, represents a step in that direction. Although we identified a number of genes-of-interest that support previously identified mechanisms of TPP-based toxicity, using the DEGs and GO/KEGG terms to predict putative phenotypes remains challenging. Toxicity-specific ontologies (OpenTox, AOP outcomes) can provide additional and complementary information, and when combined with phenotypic ontologies, offers the possibility of enhancing our prediction of phenotypic outcomes (Wang et al. 2019b). Additionally, it is necessary to continue to determine homologous relationships between zebrafish and human ontologies in order to fully evaluate the results seen in zebrafish in a human context.

Although there were significant differences between the 2 exposure paradigms, both identified nearly identical detoxification/xenobiotic-related pathways. This is unsurprising, as detoxification is an expected response to chemical challenge and may also be considered among a core set of genes/pathways expected to be seen upon chemical challenge.

## Conclusion

In this paper, we demonstrate distinct transcriptomic outcomes based on two exposure paradigms. Of particular interest, the transcriptomic results from both models identified a number of affected genes and pathways (endocrine disruption, cell cycle, detoxification) that would not be detectable through standard phenotypic observations. By combining transcriptomic data with uptake and metabolism data, we can better understand the differences and complementarity between the two protocols. As the zebrafish larval model becomes a more integrated component of chemical risk assessment, exploiting the various nuances of different exposure paradigms, such as whether or not organogenesis is included in the exposure period, will strengthen the information obtained from this early life-stage zebrafish model.

## Supplementary Material

kfaf124_Supplementary_Data
