# Supplementary material for: Testing the effects of two different zebrafish exposure paradigms on transcriptomic-based chemical risk assessment using the flame retardant triphenyl phosphate
Source: Toxicol Sci. 2025 Sep 9;208(1):176–85. doi: 10.1093/toxsci/kfaf124 (PMC12599871; doi:10.1093/toxsci/kfaf124)
Supplement: kfaf124_Supplementary_Data [file kfaf124_supplementary_data.zip › kfaf124_Supplementary_Data/toxsci-25-0339-File001.docx]

Supplementary Data.

Supplemental Table 1. Selected parameters of STAR alignment and abundance quantification. See below.

Supplemental Figure 1. Mode calculations for TPP using the ZET and GBT exposure paradigms. See below.

Supplemental file 1 – TPP ZET EC_20_ vs CC_20_ DEGs using a 1.5-fold change cutoff and a padj (q) < 0.05.

Supplemental file 2 – TPP GBT EC_20_ vs CC_20_ DEGs using a 1.5-fold change cutoff and a padj (q) < 0.05.

Supplemental file 3 – ClueGO results for ZET, Common/Shared and GBT DEGs.

|  | ZET | GBT |
| --- | --- | --- |
|  | Avg | Avg |
| Number of input reads | 14722211 | 15272733 |
| UNIQUE READS: |  |  |
| Uniquely mapped reads number | 12108410 | 12853394 |
| Uniquely mapped reads % | 82.52 | 84.16 |
| Average mapped length | 123.02 | 122.95 |
| Number of splices: Total | 5852704 | 5100326 |
| Number of splices: Annotated (sjdb) | 5739678 | 4997183 |
| Mismatch rate per base, % | 0.51 | 0.46 |
| Deletion rate per base | 0.0004 | 0.0005 |
| Deletion average length | 2.56 | 2.52 |
| Insertion rate per base | 0.0002 | 0.0003 |
| Insertion average length | 2.18 | 2.19 |
| MULTI-MAPPING READS: |  |  |
| Number of reads mapped to multiple loci | 1999127 | 1926114 |
| % of reads mapped to multiple loci | 13.3 | 12.6 |
| UNMAPPED READS: |  |  |
| % of reads unmapped: too many mismatches | 0 | 0 |
| % of reads unmapped: too short | 3.58 | 2.65 |
| % of reads unmapped: other | 0.3 | 0.32 |
| CHIMERIC READS: | 0 | 0 |

Supplemental Table 1. Selected parameters of STAR alignment and abundance quantification.


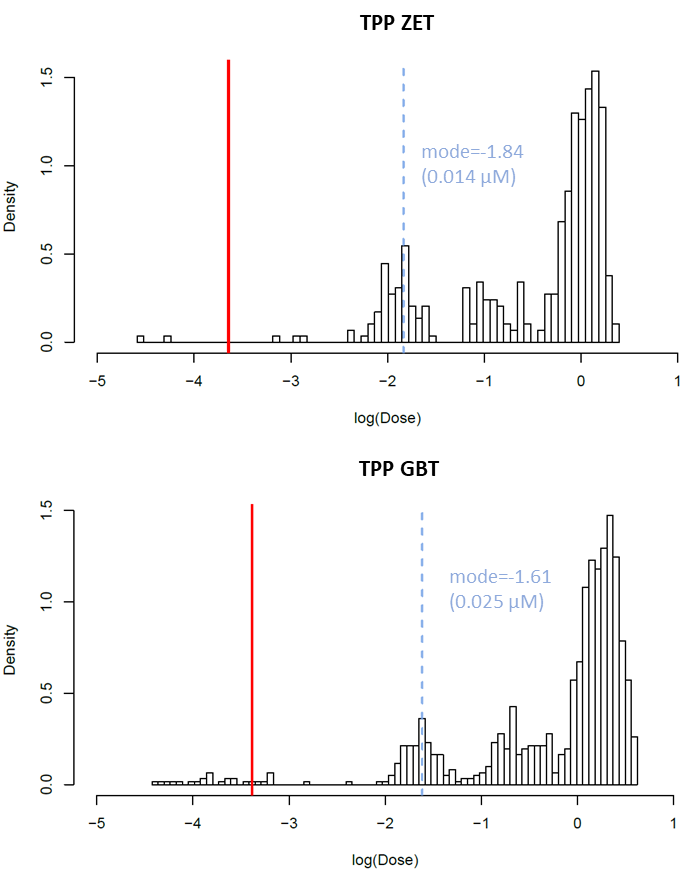


Supplemental Figure 1. Histograms of mode values obtained from BMDExpress 2.3. Light blue hashed line indicates the first calculated mode, as well as the corresponding concentration.
